# Lynch syndrome: still not a familiar picture

**DOI:** 10.1186/1477-7819-6-21

**Published:** 2008-02-20

**Authors:** Frederik J Hes

**Affiliations:** 1 Center for Human and Clinical Genetics (CHKG), Department of Clinical Genetics, Leiden University Medical Center (LUMC), RC Leiden, The Netherlands

## Abstract

**Background:**

Germ line mutations in mismatch repair genes underlie Lynch syndrome and predispose carriers for colorectal carcinoma and malignancies in many other organ systems.

**Case presentation:**

A large Lynch syndrome family with 15 affected family members and  involvement in 7 organs is reported. It illustrates a lack of awareness  and knowledge about this hereditary tumor syndrome among doctors as well  as patients. None of the described family members underwent presymptomatic screening on the basis of the family history.

**Conclusion:**

Hereditary features, like young age at diagnosis, multiple tumors in multiple organs and a positive family history, should lead to timely referral of suspected cases for genetic counseling and diagnostics. For Lynch syndrome, these features can be found in the Amsterdam and Bethesda criteria. Subsequently, early identification of mutation carriers might have diminished, at least in part, the high and early morbidity and mortality observed in this family.

## Background

Colorectal carcinoma (CRC) is an important cause of cancer-related death in the Western world. The lifetime risk is about 5% and is rising [1]. Currently, about 5% of all CRC cases can currently be explained by known inherited tumor syndromes. The most common of the known CRC predisposing syndromes is Lynch syndrome (previously also annotated as hereditary non-polyposis colorectal cancer; HNPCC) which is characterized by the development of CRC, endometrial cancer and various other cancers [2]. This tumor syndrome is caused by a mutation in one of the mismatch repair (MMR) genes: *MLH1*, *MSH2*, *MSH6 *or *PMS2*. Tumors observed in Lynch syndrome families are diagnosed at an unusual early age and may be multiple. The MMR-defect leads to instability at microsatellites of tumor-DNA that is called microsatellite instability (MSI). Subsequently, with immunohistochemical (IHC-) analysis using antibodies against the four MMR-proteins, loss of protein expression of the causative gene can be demonstrated. In order to standardize clinical and basic research the Amsterdam criteria were first published in 1991 and revised in 1999 [3,4]. In 1997, the Bethesda guidelines were developed to select patients that should be tested for MSI and IHC. These guidelines were revised in 2004 [5,6]. The revised Amsterdam criteria and Bethesda guidelines are shown in Table 1. These guidelines have enabled the recognition of vast numbers of affected families, and germline mutation analysis of the MMR-genes has led to identification of many (asymptomatic) family members at risk for Lynch syndrome. However, this case report illustrates a lack of awareness about this hereditary tumor syndrome among doctors as well as patients.

**Table 1:** Amsterdam criteria II and revised Bethesda guidelines.

Amsterdam criteria II
There should be at least three relatives with colorectal cancer (CRC) or with a Lynch syndrome associated cancer: cancer of the endometrium, small bowel, ureter or renal pelvis.
- one relative should be a first-degree relative of the other two; - at least two successive generations should be affected, - at least one tumor should be diagnosed before the age of 50 years, - FAP should be excluded in the CRC case if any, - tumours should be verified by histopathological examination.
Revised Bethesda guidelines
1. CRC diagnosed in a patient aged <50 years.
2. Presence of synchronous, metachronous colorectal, or other Lynch syndrome-related tumours*, regardless of age.
3. CRC with MSI-high phenotype diagnosed in a patient aged < 60 years.
4. Patient with CRC and a first-degree relative with a Lynch syndrome-related tumor, with one of the cancers diagnosed aged <50 years.
5. Patient with CRC with two or more first-degree or second-degree relatives with a Lynch syndrome-related tumor, regardless of age.

*Lynch syndrome related tumours include colorectal, endometrial, stomach, ovarian, pancreas, ureter, renal pelvis, biliary tract, and brain tumours, sebaceous gland adenomas and keratoacanthomas and carcinoma of the small bowel

## Case presentation

**Figure 1:**
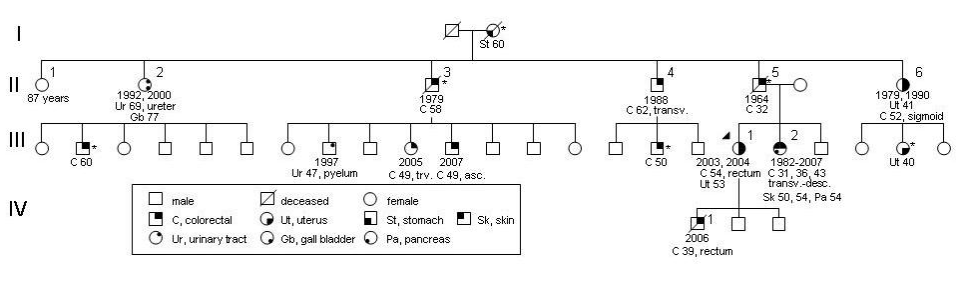
**Pedigree of a Lynch syndrome family, showing organ systems and age of diagnosis, see legend.** Year, year of diagnosis/diagnoses; asterisk, anamnestically obtained information; urinary tract, urothelial carcinoma of renal pelvis or ureter; skin, keratoacanthoma; asc., ascending colon; transv., transverse colon; desc., descending colon.

In November 2006, a 56-year old woman (III-1 in pedigree, Figure 1) visited our clinic for genetic counseling because she was worried about the many cases of cancer that had occurred in her family. The direct reason for her visit was the recent death of her 39-year old son (IV-1) with a symptomatic, and already metastasized, rectal adenocarcinoma. The counselee had been diagnosed with an endometrial and a sigmoid carcinoma at age 53- and 54-years old, respectively. She reported her overwhelming family history, which easily fulfilled the criteria that enable selection of families that are at risk for Lynch syndrome (Table 1). Subsequent IHC-analysis on archival tumor material of her sigmoid carcinoma demonstrated abrogation of the MSH2 and MSH6 proteins, which is typically associated with a germline *MSH2 *mutation. Multiplex ligation-dependent probe amplification (MLPA), in DNA extracted from peripheral lymphocytes, identified an entire *MSH2 *gene deletion (exons 1–16) and confirmed the diagnosis of Lynch syndrome.

In retrospect, some doctors had indeed signaled noteworthy features in this family. First, in 1979, a gynecologist who was treating patient II-6 consulted a colleague about the very early onset of endometrial carcinoma. His colleague reassured him at that time that the age at presentation, 41 years old, was not in fact very rare. Second, in 2005, a gastroenterologist spoke of possible HNPCC in patient III-2, who was diagnosed with carcinoma of the papilla of Vater after she had developed three separate colon carcinomas, but no further action was taken. Third, in 2006, an oncologist treating patient IV-1 suggested MSI testing on tumor material after his mother (III-1) had expressed her concern about the family history, but did not proceed.

## Discussion

This family is a fine example of the plethora of tumors that may occur in Lynch syndrome and demonstrates why the term Lynch syndrome is preferred nowadays over HNPCC (hereditary non-polyposis colorectal cancer), which only refers to CRC. The organ involvement in this family included seven organ systems: colon, uterus, skin, stomach, urinary tract, pancreas and hepatobiliary system. The manifestation of keratoacanthoma in this family enabled a sub-classification to Muir Torre syndrome (MTS). MTS is a variant of Lynch syndrome and germline mutations in the three main Lynch syndrome genes (*MLH1*, *MSH2 *and *MSH6*) have been identified in MTS families [7,8]. Keratoacanthoma should be regarded as one of the tumors that lie in the constellation of Lynch syndrome but their manifestation could depend on modifier genes and/or environmental factors.

This case report shows a considerable delay in diagnosing Lynch syndrome which negatively influenced the management of many family members. None of the family members underwent presymptomatic screening on the basis of the family history, while clinical surveillance has been shown to decrease mortality in Lynch syndrome families [9]. Remarkably, in none of the medical reports we obtained was a family history reported extending further than first-degree relatives.

## Conclusion

This family clearly illustrates a lack of awareness about a hereditary tumor syndrome among doctors as well as patients. In general, it is prudent to be aware of classic hereditary features, like young age at diagnosis, multiple tumors in multiple organs and a positive family history, and to refer suspected cases for genetic counseling. More specifically, these features can be found in the Amsterdam and Bethesda criteria (Table 1). Subsequently, the identification of at-risk persons will optimize the timing and efficiency that surveillance and treatment are carried out.

## Abbreviations

CRC: colorectal cancer; HNPCC: hereditary non-polyposis colorectal cancer; IHC: immunohistochemistry; MSI: microsatellite instability; MLPA: multiplex ligation-dependent probe amplification; MTS: Muir Torre syndrome

## Competing interests

The author(s) declare that they have no competing interests.

## Authors' contributions

FJH contributed to conception and design, acquisition of data, analysis and interpretation of data. He also drafted the manuscript and gave final approval of the version to be published.
